# Eighth International Course on Dengue and Dengue Hemorrhagic Fever: Still a Menace to the Public Health of the Americas

**DOI:** 10.3201/eid0905.051234

**Published:** 2003-05

The Pan American Health Organization (PAHO)/World Health Organization (WHO) Center for Viral Diseases and PAHO/WHO Center for Medical Malacology and Vector Control of the “Pedro Kourí” Tropical Medicine Institute (IPK) in Havana, Cuba, the Ministry of Health, PAHO, and TDR/WHO announce the Eighth International Course on Dengue and Dengue Hemorrhagic Fever: Still a Menace to the Public Health of the Americas. On the 170th anniversary of the birth of Carlos J. Finlay, the Cuban scientist who discovered the transmitting agent of yellow fever, the *Aedes aegypti* mosquito, vector control continues to be the only alternative available to stop the spread of the dengue. The 8th biannual International Course on Dengue will be held in Havana, Cuba on August 11 to 22, 2003.

The objective of the course is to provide a forum for participants and presentations by lecturers who specialize in virology, epidemiology, vector control, immunology, sociology, and medical care and have experience with dengue fever and dengue hemorrhagic fever (DHF). Participants will be able to review up-to-date facts about dengue.

All presentations will be in Spanish. Practical sessions will be divided into three groups (laboratory diagnosis; entomology, vector control, and community participation; and clinical and pathologic aspects) according to the professional profile of attendants. Topics will include dengue and DHF in the Americas, new viruses in old areas and old viruses in new areas, West Nile virus in the Americas, epidemiology of dengue, clinical and therapeutic aspects of dengue and DHF, healthcare organization in emergency situations, laboratory diagnosis, molecular biology of dengue viruses, molecular evolution/epidemiology of dengue, bioinformatics and dengue, immunopathogeny and physiopathology, T-cells responses, vaccine development update, and social sciences, vector control, and dengue prevention.

Applications should be sent before July 1, 2003, to: Prof. Maria G. Guzman, Instituto Pedro Kouri, Autopista Novia del Mediodia, Km. 6 P.O. Box Mnao 13, Ciudad Habana, Cuba; fax: 53-7-2020460; telephone: 53-7-246051 or 53-7-2020633; email: lupe@ipk.sld.cu. Applications should include the name and address of applicant, telephone and fax numbers, and email; a summarized curriculum vita; and the practical session of preference. Registration is $1,000 (U.S), which includes registration, course materials, welcome cocktail, and farewell dinner).

For additional information visit the Web site: http://www.ipk.sld.cu/eventosipk/curso-dengue1.htm (English) or http://www.ipk.sld.cu/eventosipk/curso-dengue1.htm (Spanish).

